# Hidden peril: Large ciliary body melanoma imitating cataract in a cardiac patient

**DOI:** 10.22336/rjo.2025.18

**Published:** 2025

**Authors:** Sonali Vinay Kumar, Manoj Gopal Madakshira, Vinay Kumar, Natasha Vinay Kumar, Sourabh Kumar

**Affiliations:** 1 (Ophthalmology), Command Hospital Eastern Command, Kolkata, West Bengal, India; 2 (Pathology), Command Hospital Eastern Command, Kolkata, West Bengal, India; 3 (Anatomy), JIS School of Medical Science and Research, Howrah, West Bengal, India; 4 Department of Medicine, Sri Devaraj Urs Medical College, Kolar, Karnataka, India

**Keywords:** large, delayed diagnosis, ciliary body melanoma, cataract, metastasis, enucleation, IOL = Intraocular lens, UBM = ultrasound biomicroscopy, MRI = magnetic resonance imaging, PET = positron emission tomography, HPE = histopathological examination

## Abstract

Ciliary body melanoma is a rare and aggressive ocular tumor that often presents with nonspecific symptoms, leading to delayed diagnosis. This report presents the case of a 43-year-old male with a history of ischemic heart disease who presented with diminished vision in the left eye, initially attributed to a cataract. Despite prior evaluation, no sign of malignancy was detected, and the patient was advised to proceed with cataract surgery. The patient visited our center for a second opinion, where a comprehensive evaluation identified a large ciliary body melanoma extending into the anterior chamber. Management was challenging due to the patient’s cardiac condition, as anticoagulant therapy could not be discontinued. Enucleation was performed to mitigate the high risk of hematogenous metastasis associated with the tumor’s rich vasculature, ciliary body contraction, and potential extension through emissary canals. Histopathology confirmed the diagnosis of spindle cell melanoma. This case highlights the diagnostic complexities of ciliary body melanoma, which can masquerade as a common condition like cataract, emphasizing the need for vigilance in atypical presentations and the importance of thorough evaluation to avoid misdiagnosis. It also underscores the challenges in managing such tumors in patients with significant systemic comorbidities, requiring a multidisciplinary approach for optimal outcomes.

## Introduction

Ciliary body melanoma is an uncommon ocular tumor, accounting for about 6% to 12% of uveal melanoma. Yet, it poses a significant risk due to its potential for local invasion and distant metastasis [1]. The diagnosis of ciliary body melanoma is challenging on account of subtle presentation with symptoms that may overlap with more innocuous conditions like cataract or glaucoma [2]. It often goes undetected in its early stages due to its location behind the iris, making its presentation deceptive [3,4]. A high degree of clinical suspicion and meticulous examination are mandatory to establish the diagnosis. Herein, we present a large case of ciliary body melanoma in a cardiac patient who was initially diagnosed as a case of cataract and was advised cataract removal with intraocular lens (IOL) implantation. The patient sought a second opinion at our center, and on thorough evaluation, the patient was found to have a sizeable ciliary body melanoma. Due to the significant size of the tumor and the high risk of metastasis, globe-sparing surgery was deemed unsuitable. As a result, we proceeded with enucleation with implant to minimize the chances of systemic spread. Moreover, the patient had been recently diagnosed with ischemic heart disease, which prevented the discontinuation of anticoagulants before the procedure. Managing the life-threatening nature of the ciliary body melanoma while addressing cardiac risk needs a comprehensive, multidisciplinary approach, which was adopted in our case.

## Case report

**Fig. 1:**
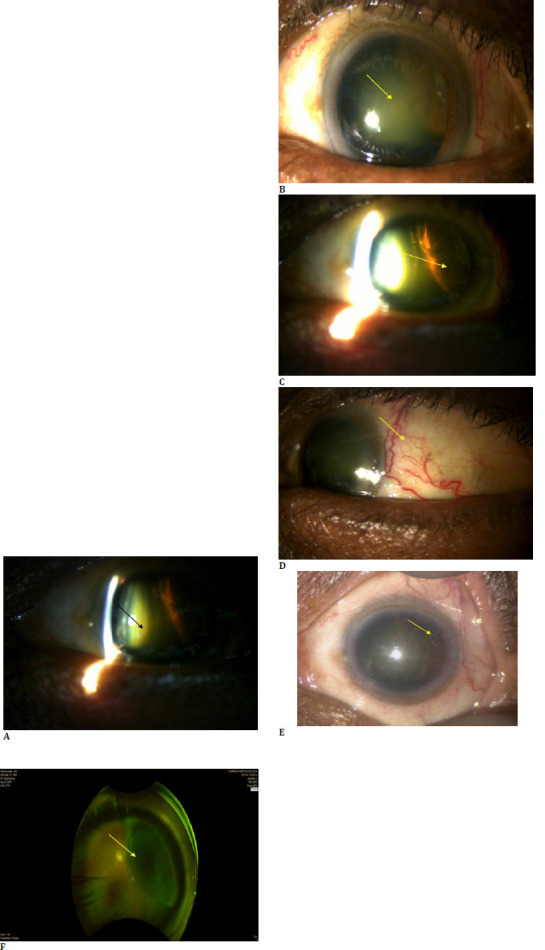
**A, B**. Slit lamp image depicting grade 2 nuclear sclerosis in the lens of left eye (black and yellow arrow); **C**. Slit lamp photograph revealing a pigmented mass behind the iris in the superotemporal quadrant in left eye (yellow arrow); **D**. Slit lamp photograph showing dilated episcleral vessel over the affected region (yellow arrow); **E**. Slit lamp depicting a subluxated lens in the respective area (yellow arrow); **F**. Clinical photograph illustrating a mass arising from the ciliary body in left eye (yellow arrow)

A 43-year-old patient presented with gradually decreasing vision in the left eye for the last 3 months. The patient did not give a history of trauma, flashes, or floaters. The patient also denied a prior history of ocular intervention. He had a history of cardiac issues, specifically ischemic heart disease, and was under regular follow-up with his cardiologist. The patient initially consulted elsewhere with similar complaints and was diagnosed with cataract, for which cataract extraction with IOL implantation was advised. Seeking a second opinion, the patient visited our center with the same symptoms. Initial ophthalmic examination showed that the best-corrected visual acuity in the right eye was 6/6; in the left, it was 6/60. The pupil was round, central, and equally reacting to light in both eyes. Intraocular pressure was within normal limits in both eyes. Slit lamp examination showed grade 1 nuclear sclerosis in right eye and grade 3 in left eye **(Fig. 1 A, B**). It also disclosed a pigmented superotemporal mass behind the iris in the left eye and a few dilated episcleral vessels over the temporal part of the same eye (**Fig. 1 C, D**). The lens was also found subluxated in the corresponding area in the left eye (**Fig. 1 E**). On indirect examination, a mass protruding from the ciliary body was observed in the left eye (**Fig. 1 F**). The superotemporal part of the retina was not visible, while the rest of the optic disc and retinal vessels were found normal. Fundus examination was unremarkable in the right eye.

**Fig. 2:**
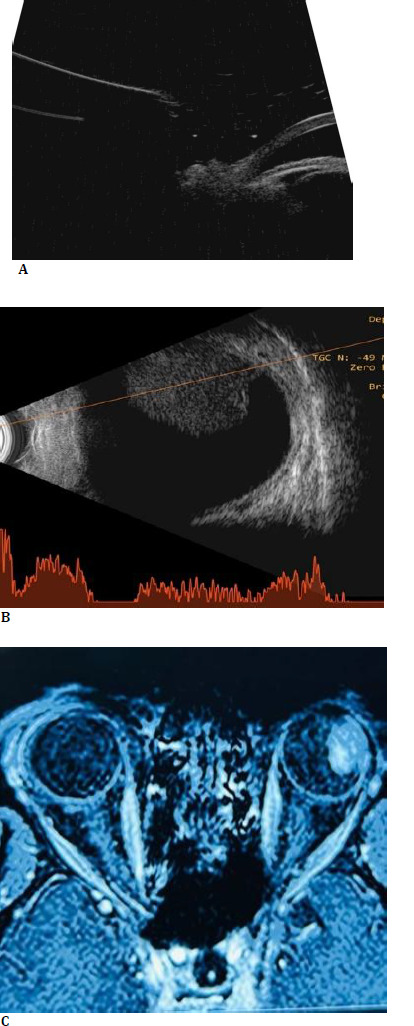
**A**. Ultrasound biomicroscopy (UBM) showing an elevated lesion in the ciliary body of the left eye; **B**. Brightness scan ultrasound image showing a dome-shaped lesion arising from the ciliary body; **C**. MRI orbit showing a lesion on the lateral aspect of the left globe

An ultrasound biomicroscopy (UBM) revealed an atypical, elevated lesion in the ciliary body of the left eye (**Fig. 2 A**). B-scan ultrasonography showed an irregular, dome-shaped lesion measuring 12 x 10 mm in size in the left eye (**Fig. 2 B**).

Magnetic resonance imaging (MRI) of the orbit and brain disclosed a well-defined lesion 14.6 x 11.4 x 9.5 mm (APXTRXCC) on the lateral aspect of the left globe. Laterally, the lesion was merging with the outer wall, and medially, the lesion exhibited polypoidal outlines. Anteromedially, it abutted the lens (**Fig. 2 C**). The lesion was hyperintense on T1W1 and hypointense on T2W1. A differential diagnosis of ciliary body melanoma was established based on clinical presentation and imaging findings.

**Fig. 3:**
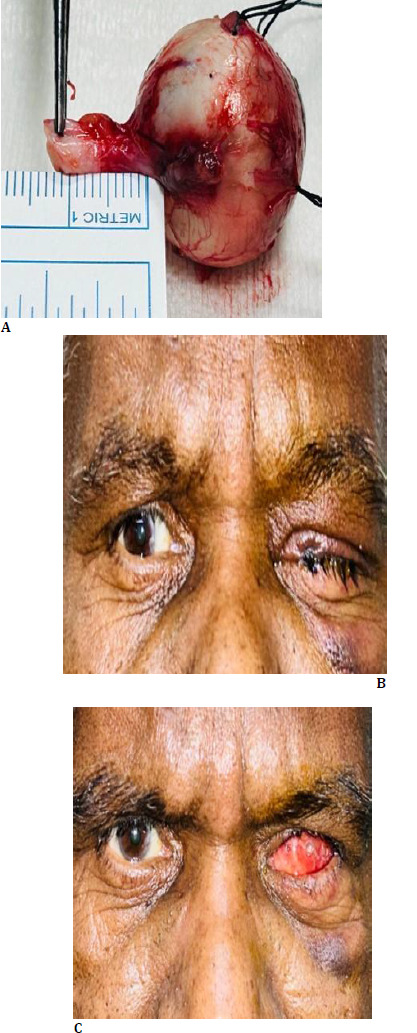
**A**. Clinical photograph depicting an enucleated eyeball with attached optic nerve; **B**. Post-operative figure showing an anophthalmic socket with tarsorrhaphy suture at one week; **C**. Post-operative figure showing an anophthalmic socket with conformer in place at one month

**Fig. 4:**
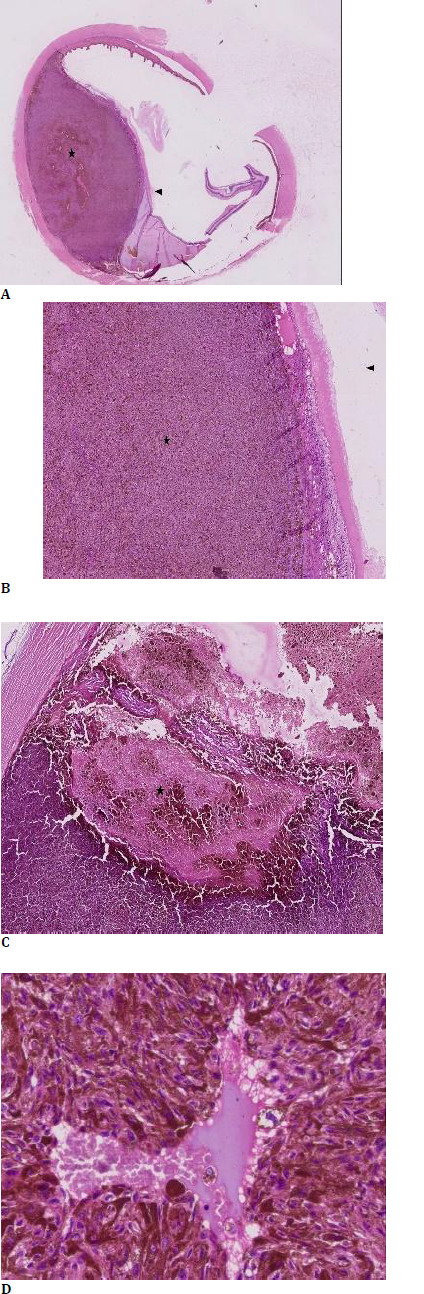
**A**. Hematoxylin and Eosin stain (1x magnification) showing a dome-shaped tumor (star) arising from the ciliary body (arrow); **B**. H and E stain (4x magnification) showing tumor (star) lifting the overlying layers of the ciliary body (arrow); **C**. Hematoxylin and Eosin stain (4x magnification) showing areas of geographic tumor necrosis are observed (star) surrounded by viable tumor cells; **D**. Grocott stain (40x magnification) highlights the spindle-shaped tumor cells having vesicular nuclei and granular black cytoplasmic pigment

A positron emission tomography (PET) scan was ordered, which showed no sign of systemic involvement because of the lesion. Serum lactate dehydrogenase (LDH) was found on the higher side (LDH-235 IU/L). As the size of the lesion was more than 14 mm in basal diameter and thickness more than 8 mm, we opted for enucleation rather than globe-sparing surgery as the risk of hematogenous metastasis is very high in ciliary body melanoma due to the rich vasculature of this anatomical region and its continuous muscular contractions. The proximity to emissary canals increases the likelihood of anterior and posterior extension of the tumor. The patient’s anticoagulation therapy added an additional layer of complexity, as stopping it before surgery could exacerbate his cardiac issues. However, continuing it could increase the risk of intraoperative bleeding. Enucleation with implant was chosen as the most viable option to prevent the risk of metastasis (**Fig. 3 A**). Blood was arranged, and topical brimonidine was started preoperatively to manage intraocular pressure and minimize the bleeding risk during surgery. The enucleated eyeball was sent for histopathological examination (HPE). HPE showed lesions composed of spindle cells and associated with necrosis (20%). The spindle cells have oval nuclei with prominent nucleoli and pale eosinophilic cytoplasm. Many of the tumor cells showed fine granular pigment. HPE suggested a feature of spindle cell melanoma (**Fig. 4 A-D**). The patient was followed up for 6 months and showed no sign of local recurrence and systemic metastasis (**Fig. 3 B**).

## Discussion

Ciliary body melanomas are extremely rare but aggressive types of uveal melanoma and, due to their hidden location, are usually present in the more advanced stage. They arise from melanocytes in the ciliary body, a part of the eye involved in aqueous production and lens accommodation [5,6]. The clinical symptoms and signs of ciliary body melanoma are often subtle and can easily be mistaken for more common ocular conditions. Patients may initially present with symptoms like blurred vision, decreased visual acuity, or a visible mass if the tumor has invaded the anterior segment. The tumor may induce changes such as cataract formation and can cause raised intraocular pressure or even secondary retinal detachment. Due to these overlapping symptoms, ciliary body melanoma is often misdiagnosed, hence delaying the appropriate intervention [7]. Ciliary body melanoma usually attains a large size before it is recognized clinically. The typical external sign that suggests this entity is dilated episcleral blood vessels (sentinel vessels) that develop over the base of the tumor. The other external sign is an epibulbar pigmented lesion, which is characteristic of transscleral extension of the tumor. It can grow posteriorly into the choroid (ciliochoroidal melanoma) or anteriorly into the anterior chamber angle and iris (iridociliary melanoma). It can infiltrate the trabecular meshwork, causing secondary glaucoma. Managing ciliary body melanoma depends on the tumor's size, location, extent, and patient factors such as age and overall health [8].

Some studies have drawn attention to the early manifestations of ciliary body melanoma, which often leads to its misdiagnosis and subsequent delay in treatment [8-10].

In our case, the tumor was overlooked in its early stages and was erroneously diagnosed as a case of cataract. It was only identified later when it had grown significantly and started protruding into the anterior chamber. Advanced imaging, such as UBM and MRI orbit, played a vital role in accurately diagnosing this entity in the present case.

The standard approach for a ciliary body melanoma typically involves options like plaque brachytherapy, local resection, or enucleation. In this case, enucleation was chosen over globe-sparing surgery due to the size of the tumor and the high risk of metastasis. For cardiac patients, especially those on anticoagulant therapy, the surgical risks are heightened. Stopping anticoagulants could exacerbate cardiac issues, while continuing them increases the risk of intraoperative bleeding. This delicate balance needed a comprehensive preoperative evaluation and collaboration between the ophthalmology, cardiology, and anesthesiology teams. Anticoagulant therapy was not discontinued to prevent any further aggravation of the patient’s cardiac condition, and enucleation was carried out in this case. Enucleation in this context was deemed the safest and most definitive option to prevent systemic spread.

## Conclusion

Although rare, ciliary body melanoma is a significant diagnostic challenge due to its nuanced presentation and potential to mimic other intraocular conditions like cataracts. A high suspicion index and appropriate imaging techniques are essential for early diagnosis and management. Treatment requires a tailored approach based on tumor characteristics and patient health, and close follow-up is necessary to detect and manage potential metastasis.
